# Balancing the playing field: collaborative gaming for physical training

**DOI:** 10.1186/s12984-017-0319-x

**Published:** 2017-11-20

**Authors:** Michael Mace, Nawal Kinany, Paul Rinne, Anthony Rayner, Paul Bentley, Etienne Burdet

**Affiliations:** 1 Department of Bioengineering, Imperial College of Science, Technology and Medicine, London, UK; 2 Division of Brain Sciences, Imperial College of Science, Technology and Medicine, London, UK; 3 Center for Neuroprosthetics, École Polytechnique Fédérale de Lausanne, Lausanne, Switzerland; 4 School of Mechanical and Aerospace Engineering, Nanyang Technological University, Singapore, Singapore

**Keywords:** Social interaction, Collaboration, Rehabilitation, Stroke, Physical exercise, Patient engagement, Exergames, Robotics

## Abstract

**Background:**

Multiplayer video games promoting exercise-based rehabilitation may facilitate motor learning, by increasing motivation through social interaction. However, a major design challenge is to enable meaningful inter-subject interaction, whilst allowing for significant skill differences between players. We present a novel motor-training paradigm that allows real-time collaboration and performance enhancement, across a wide range of inter-subject skill mismatches, including disabled vs. able-bodied partnerships.

**Methods:**

A virtual task consisting of a dynamic ball on a beam, is controlled at each end using independent digital force-sensing handgrips. Interaction is mediated through simulated physical coupling and locally-redundant control. Game performance was measured in 16 healthy-healthy and 16 patient-expert dyads, where patients were hemiparetic stroke survivors using their impaired arm. Dual-player was compared to single-player performance, in terms of score, target tracking, stability, effort and smoothness; and questionnaires probing user-experience and engagement.

**Results:**

Performance of less-able subjects (as ranked from single-player ability) was enhanced by dual-player mode, by an amount proportionate to the partnership’s mismatch. The more abled partners’ performances decreased by a similar amount. Such zero-sum interactions were observed for both healthy-healthy and patient-expert interactions. Dual-player was preferred by the majority of players independent of baseline ability and subject group; healthy subjects also felt more challenged, and patients more skilled.

**Conclusion:**

This is the first demonstration of implicit skill balancing in a truly collaborative virtual training task leading to heightened engagement, across both healthy subjects and stroke patients.

**Electronic supplementary material:**

The online version of this article (doi:10.1186/s12984-017-0319-x) contains supplementary material, which is available to authorized users.

## Background

Physiotherapy intensity is a well-recognised determinant of stroke recovery, although questions of method, timing, scheduling, etc., are still debated [1]. Video games have been highlighted as a means to increase therapy intensity, by enabling round-the-clock access to exercises, independent of professional supervision, while incentivizing through stimulating feedback. Exercise games (‘exergames’) can replicate aspects of conventional physiotherapy such as repetitive joint stretches, functional manipulation, difficulty adaptation, while manipulating motivational and cognitive variables [2–4]. Incorporation of these factors can increase therapy efficiency, and facilitate skill transfer to real world function [5].

Recently, virtual therapy involving two or more players has been proposed as a means of further increasing intrinsic motivation, engagement and social inclusion [6–12]. By promoting social interaction alongside entertainment, the appeal of gamification can be extended to a broader audience who may otherwise be disinterested due to age, impairment, cognitive or experiential issues. Furthermore, playing with another patient, a carer, or a relative at the hospital or at home can prevent patient isolation.

Compared to single-player training games, multiplayer games are more engaging, with the level of impact depending partly upon participant personality traits [6, 12]. To date, the majority of multiplayer rehabilitation exergames do not elicit true motor interactions, in the sense of each individual’s performance being directly influenced by the other. For example, it is common for multiplayer games (e.g. [6, 13]) to divide goals into subtasks that can be completed independently by the players (whether or not simultaneously), without the performance of one player being influenced by the other(s) [14, 15]. By contrast, visuomotor learning paradigms that physically connect two subjects, can enforce inter-subject interactions, evident as a performance benefit not only for the weaker, but also the stronger partner (as shown in healthy populations) [16]. This occurs through models of motor planning based upon a connected partner’s intentions, communicated both visually and haptically [17]. However, to provide physical-coupling between two subjects requires the integration of a complicated robotic system which is not broadly applicable during home-based rehabilitation, an area where technology and gamification can make a significant difference. Therefore, this study aims to virtualise many of the latent aspects of a physical connection, using visual-coupling and task design alone, to enable more accessible and cost-effective sensor-based systems (e.g. MusicGlove [18]) and ultimately for these systems to benefit from such strategies. Despite removing the ability to physically assist patients, by defining a new paradigm in virtual human-human interaction, we aim to promote better (force) control and player engagement, regardless of any underlying skill mismatch between the participants. This should also prevent natural motor ‘slacking’ a common issue when using active assist devices. By utilising sensor-based technology which are both sensitive and work on functional movements (e.g. contributing to activities of daily living), more efficient rehabilitation can be achieved, as active participation from the impaired limb is required if a patient is to ultimately recover volitional and functional movement.

A significant issue in the design of multiplayer games, particularly amongst disabled users, is how to permit differences in skill-levels between players, and allow for effective gameplay, participation and enjoyment by all players [3, 19, 20]. If player abilities are not correctly balanced, the challenge will be too high and quickly lead to frustration for the less skilled player; while the more skilled player will not be challenged and is likely to become bored. A related concern is how to design a multiplayer game that inhibits natural slacking behaviour, in which one player (usually the less-skilled one) becomes disengaged, even though the overall game performance is maintained [21, 22]. For example, in the cooperative-mode of the classic pong game [11, 13], interaction between the partners is not required as the game can be completed with only one player active (e.g. the skilled player can score points even if the less skilled player misses). Although multiplayer functionality can promote exergame engagement, it is unclear which type results in the most effective interaction, especially for less-able subjects who are in danger of ‘falling behind’.

Inter-player relationships broadly fall into one of four types of human-human interaction [14, 15].

- **Co-activity** characterised by a divisible task that either player can complete independently.
- **Competition** each player interacts with the partner to fulfil their own goal and ultimately prevent the other player fulfilling their aim.
- **Cooperation** the players work together to complete the task but have different roles (such as assistance i.e. master-slave, or educator-student).
- **Collaboration** the interacting players are assigned the same role and need to work together to complete the task.

Previous rehabilitation games involving multiple players have focused on either co-active or competitive ([6, 11, 13]) types of interaction^1^. However, collaborative and cooperative interactions have several beneficial properties which can further promote motivation [10, 23]. These include: i) players needing to work together to achieve a common goal, thus promoting positive teamwork; ii) neither player is able to slack as the task is not divisible; iii) communication between players can help complete the task and promotes increased social inclusion. Additionally, in the case of collaboration, iv) having similar roles and task-goals enables consensual interaction, potentially empowering the patient by not a priori assigning them the role of the ‘learner’, and ultimately reducing the need for explicit instructions (with the latter requiring linguistic and cognitive aptitude). Given the theoretical advantages of collaboration, relative to other forms of inter-player interactions, we describe here a novel physical-training, social-gaming software, that embodies true collaboration. The aim of this proof-of-concept study is to elucidate on the effects of dual-player collaboration on human-human performance based on individual sensorimotor control whilst interacting in a visually-coupled task (i.e. there is no haptic or physical coupling between the dyads). This is compared with an equivalent single-player version, alongside user-experience, in both able and disabled subjects^2^.

## Methods

### Balancing act: multiplayer collaborative gaming

A motor-training paradigm was designed such that two subjects could train concurrently, while interacting and skill-sharing, regardless of baseline differences in subject ability. The two players may be, for example, a therapist and a disabled patient, two patients with differing disabilities, or even a sports-person and their coach.

The following characteristics were considered to be advantageous for an efficient training game:

- **Simple** A simplified game, both in terms of strategy and graphics (e.g. 2D), allows individuals to focus on gameplay and sensorimotor control, while reducing distraction.
- **Dynamic** A continuously changing task places higher demands on motor control, and encourages visuomotor coordination, sustained attention, and player engagement.
- **Multifaceted metrics** Performance feedback to subjects in an immediate and readily-comprehensible fashion (e.g. points collected), can motivate them to achieve task goals and maintain practice [24]. Concurrent information can be extracted to quantify how subjects interact, and examine the (social) strategies deployed.

Secondly, we considered these characteristics to be necessary to foster true social interaction:

- **Interactive** The partners are virtually connected (visually and/or haptically), with their actions influencing each other’s behaviour. This promotes social interaction and may motivate training.
- **Collaborative** The two players contribute equally to achieving the task goals, thus enhancing positive social interaction.
- **Locally redundant** Redundant control refers to the ability of one partner helping the other achieve a common goal (e.g. a therapist supporting a cup being lifted by a patient). However, to avoid slacking or complacency, which prevents learning, redundancy should be local, meaning the task cannot be achieved by only one of the participants alone.

It was hypothesised that these features should, i) make the task achievable by impaired individuals who could not succeed alone, and ii) increase the difficulty for the better performer by having to compensate for the worse performer. Furthermore, we expect engagement to increase through inter-subject interaction [4], because of greater task assistance and achievement (for the inferior partner), and being challenged and/or requiring altruistic behaviour (from the superior partner).

### Example embodiment: Balloon Buddies^TM^

#### Game description

**Fig. 1:**
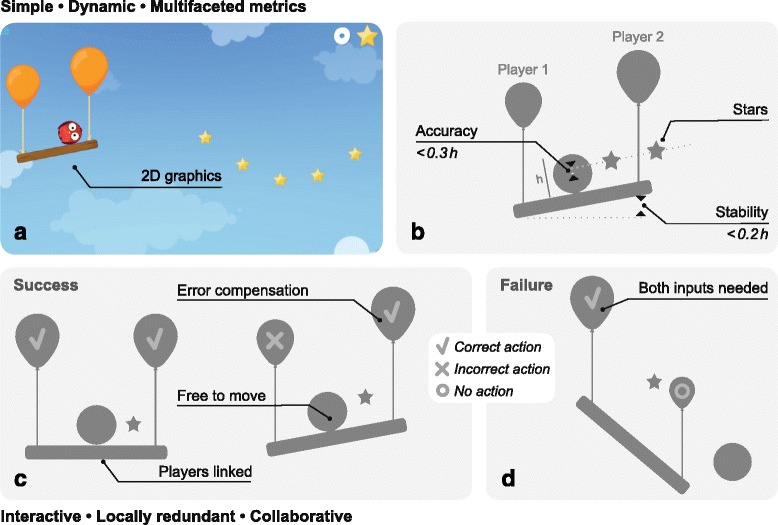
Overview of the Balloon Buddies^TM^ game. **a** A screenshot from the game, **b** the three performance metrics defined within the game: number of collected stars, accuracy (based on the distance between the buddy centre and the ideal trajectory), which should be > 30% of the buddy’s diameter *h* and stability (based on the distance between the two ends of the beam), which should be > 20% of the buddy’s diameter *h*. **c**, **d** Different scenarios are shown highlighting the local redundancy of the game, i.e. errors can be accommodated (**c**), but neither player can slack as both inputs are required (**d**)

Based on the above properties, a **two-player game** whose features could facilitate and motivate physical training was created. Figure 1 shows an overview of the game, which comprises a dynamic balance, represented by a ball on a horizontal beam, developed within a 2D physics simulation engine. The ball is represented by a circular sprite (the ‘buddy’), which is subject to physical forces, and is free to roll across the top of a rigid beam or roll off the beam subject to gravity and frictional forces. The beam is lifted at each of its ends by balloons controlled by each player, with the 2D kinematics of the beam described by

$$\left[ \begin{array}{l} h\\ \theta \end{array} \right] =\left[ \begin{array}{c} \frac{y_{1}+y_{2}}{2}\\ \arcsin\left(\frac{y_{2}-y_{1}}{L_{\text{beam}}}\right) \end{array} \right] $$

where *y*_1_ and *y*_2_ are the height of each end of the beam respectively, *L*_beam_ is the length of the beam, *h* is the height of the beam centre and *θ* is the angular position of the beam. Upward movement of each balloon is controlled by a player varying their power-grip force, applied via a digital force transducer. The more grip force applied, the higher the balloon rises. Downward movement occurs passively by relaxing the grip, in conjunction with gravity. The grip transducer used here is compliant, highly sensitive, and interacts wirelessly with a standard Android tablet [25–27]. Prior to gameplay, the software is calibrated based on the maximum power-grip ability of the user.

The vertical translation *y*_*i*_ applied to a specific balloon is driven by the calibrated force $\hat {F_{i}}$ from player *i*∈{1,2}, according to

$$c\dot{y_{i}} + y_{i} = k\hat{F_{i}}, \quad i \in \{1,2\} $$

with force visualised through the balloon’s inflation (Fig. 1). Smooth game dynamics is ensured by stiffness (*k*) and damping (*c*) terms, controlling sensitivity of the position to force and smoothness of the control, respectively. For healthy subjects *k*=1, while for patients *k*=1.8. For both groups, *c* is not defined a priori, and is instead tuned by the software to ensure that the dynamics are critically damped and have a fixed settling time (*t*_*s*_=0.09 s for healthy and *t*_*s*_=0.25 s for patients)^3^. The game and associated graphical elements are presented in Fig. 1 a. During gameplay, the whole platform scrolls at a constant speed (*v*≈22 mm/s) horizontally. All parameters (*k*,*t*_*s*_,*v*) were chosen through initial testing, using independent groups of healthy and patient subjects, based on a subjective trial-and-error procedure.

The primary aim of the game is to vary the height of the beam so that the buddy matches a moving target height. A secondary aim is to keep the buddy from rolling off the beam, requiring players to keep the beam horizontal. Players need to simultaneously control the height and inclination of the beam using their combined inputs. The target height is represented by a specified trajectory, shown as stars, which are ‘collected’ by colliding them with the buddy. Star collection results in the visually-presented game score incrementing and is accompanied by positive auditory feedback. If the buddy falls from the beam, it is inactivated for three seconds, before reappearing and dropping onto the beam. During this period, it is not possible to catch stars, thus resulting in a lower final score. For healthy subjects, the target trajectory was described by a pseudo-random function, *y*= sin(0.15*x*)+ sin(*x*)+0.5 sin(0.6*x*), where *x* is the horizontal translation, with similar functions selected in previous motor learning studies to ensure random but smooth trajectories [28]. To make it easier for patients, a predictable sinusoidal target trajectory, *y*=1.5 sin(0.5*x*), was employed^4^.

Independently of us, Vanacken et al. have previously introduced a similar ball-balancing task (‘Balance pump’) as a mini-game within their virtual rehabilitation solution targeting multiple sclerosis [23]. The main differences are, (a) their study did not define or explore the implicit skill balancing nature of the elicited interaction, (b) they utilised arbitrarily placed static targets, with no time constraints, and (c) we define a multidimensional scoring system including both performance and motor control measures. We believe (b) is more likely to lead to sequential interaction rather than continuous balanced collaboration as elicited by the smooth continuous trajectory of moving targets which is used in our study. Moreover, by defining additional measures (c), such as stability, it allows patients to achieve targets regardless of their ability to just hit stars.

In order to determine the effectiveness of dual-player functionality, a **single-player mode** of Balloon Buddies^TM^ was created. This differs from the above in that the only input is player-mediated grip-control to the left balloon. The right balloon automatically follows the ideal trajectory, independently of the player’s actions.

#### Game properties

The game described satisfies the desired properties for a physical-training game, outlined in the previous section. It has **simple rules**, and uses uncomplicated, intuitive 2D graphics, with minimal distractions. Visual cues and feedback are overlaid onto the buddy system so as to avoid saccades. For example, the buddy’s eyes close (to indicate ‘sleeping’), when the beam is horizontal and buddy is stable. The grip-force applied by each subject is depicted in realtime, by the size of each balloon. The task is **dynamic**, in that the buddy is free to move continuously (vertically or rolling horizontally) subject to ‘physical’ forces (e.g. gravity, drag, friction). Several **metrics** have been defined, including number of stars collected, accuracy of trajectory pursuit, and stability of the beam (Fig. 1 b).

Of particular relevance here are the game’s social-interaction features. Thus, the paradigm is **interactive**, with players connected by the beam such that their collective actions have consequences on both the buddy and one another. For example, Fig. 1 c and d highlights that if either one of the players perform poorly (by either under- or overshooting), for a continuous period, this will lead to the buddy rolling off the beam, unless the other player takes corrective actions e.g. by matching their grip-force. Additionally, the task is **collaborative** as the players must work together to collect stars. Each player is assigned the same role, i.e. controlling the height of the beam, without a defined leader. Finally, as Fig. 1 c highlights, the task is **locally redundant** in that the buddy and beam system can tolerate intermittent mistakes whilst maintaining performance. For instance, even if one player falls behind in their trajectory pursuit, the other player can perform a compensatory manoeuver enabling star capture, before returning the balloon to stabilise the beam (Fig. 1 c). However, such compensation is achievable only within a small range of poor performances, hence inhibiting slacking behaviour. This local redundancy enables intrinsic skill balancing within the game without requiring an additional individual skill matching procedure. However, the global difficulty level, affecting both partners, can be adjusted, for instance between healthy-healthy and patient-expert dyads, by specifying different trajectories and/or system parameters.

#### Game validation

**Fig. 2:**
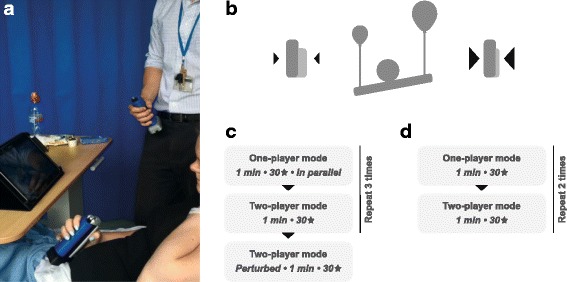
Experimental Description. **a** Hemiparetic patient and healthy expert playing the game on a tablet by controlling a grip transducer; **b** grip force controls balloon size and height; **c** healthy-healthy experimental protocol; **d** patient-expert experimental protocol

In order to assess the versatility of the software, and to see if impairment affects collaborative behaviour: i) pairs of healthy subjects, and ii) hemiparetic stroke survivors interacting with a single healthy expert subject, were tested. Figure 2 gives a general overview of the healthy-healthy and patient-expert experiments performed.

**Study 1: healthy-healthy experiment**

**Participants:** Healthy, right-handed subjects without arm disability or cognitive impairment, were recruited and consented. Handedness was assessed by the Edinburgh Handedness Inventory (EHI). Subjects were paired randomly into dyads for dual-player game participation.

**Protocol** (Fig. 2 c): Initially, the two participants played the single-player mode (A) on separate tablet-PC screens, (30 stars, 1 min). They then played the dual-player game (B) on a common tablet using constant game parameters. This play order was repeated thrice (i.e. ABABAB). Following this, they played an additional dual-player game where the control of the left player was perturbed (i.e. an increase in their sensitivity). The aim of this was to explore the effects on collaboration, when increasing the difficulty for one of the partners. Subjects were not told about this change and used their right hand for each trial. Participants were requested to refrain from talking or gesturing to each other during gameplay, so as to reduce the possibility that interactions occurred because of factors unrelated to gameplay. At the end of the experiment, participants were provided with questionnaires probing engagement and user experience (Appendix (A) Questionnaires).

**Study 2: patient-expert experiment**

**Participants:** Consecutive stroke patients with arm weakness were screened over 3 months at Imperial College NHS Healthcare, within 2-weeks of presentation. Exclusion criteria were: 1) cognitive impairment (Mini-Mental State Examination < 27), 2) premorbid arm disability, or dependency (modified Rankin Score > 2), 3) comprehension difficulty, 4) visual impairments, 5) arm pain, 6) significant co-morbidities, 7) subsequent MRI failed to confirm stroke. No distinction was made between haemorrhagic or ischaemic stroke. Patients were assessed using the Fugl-Meyer Upper Extremity (FMUE: 0-66 scale), and short form of the Fugl-Meyer (S-FM: 0-12 scale), Edinburgh Handedness Inventory (EHI), and Hospital Anxiety and Depression Scale (HADS). Approval for the study was given by the South East Coast Research Ethics Committee and all participants signed an informed consent form prior to any study-related procedure. Each patient was paired with the same healthy expert subject (right-handed male, 25 years old). This healthy subject spent two hours playing the single-player game prior to the study, which was long enough to have a stable performance over the patient-expert trials and is highlighted by their average single-player score. In this paper we denote this trained, healthy individual as ‘expert’, and use this label to differentiate this healthy subject from the novice healthy subjects which participated in Study 1.

**Protocol** (Fig. 2 d): Patients first played in single-player mode (A), followed by dual-player mode (B) alongside the healthy expert. This order was repeated twice (i.e. ABAB design). Fewer repetitions occurred in this protocol than the healthy-healthy protocol in order to limit patient fatigue. During dual-player games, verbal communication was permitted between patients and expert. All trials were played with the impaired hand by patients, and right-hand by the expert. Calibration of the handgrip-control function relative to the patient’s maximum grip-force was conducted prior to each game. To reduce the level of challenge for the patient-expert dyads, game dynamics were also simplified, by adjusting friction, angular drag of the buddy, control sensitivity (*k*) and using a simple sine-wave trajectory (see “Game description” section for details).

### Data analysis

#### Performance metrics

The following game-specific performance measures (see Fig. 1 b) were defined. All metrics were used to compare subjects performance between task conditions (i.e. single-versus-dual player modes).

1. **Nr. of stars collected:** A star is ‘collected’ when any part of the buddy diameter contacts any part of a passing star. This is a gross indicator of players’ ability to track the target trajectory, and is presented to subjects in real-time, as a cumulative score in the top right corner of the screen.
2. **Accuracy:** Computed as the percentage of time-frames in which the centre of the buddy lies within a narrow vertical margin (< 30% of the buddy’s diameter) of the reference trajectory (line connecting midpoints of stars). Whilst correlated with the ‘nr. of stars collected’, ‘accuracy’ represents finer control, and is a more challenging metric to achieve a high score on, as subjects can collect stars without being very accurate. Accuracy was displayed to participants at the end of the trial.
3. **Stability:** Reflects the degree with which the beam is held horizontally, and is computed as the percentage of frames where the vertical difference between the two ends of the beam is less than a certain threshold (< 20% of buddy’s diameter). Compared to other metrics, it is a better indicator of partner cooperation since it requires partner matching, rather than trajectory tracking. It is also a measure of control smoothness since the trajectory can be tracked accurately even though the beam moves chaotically in a seesaw manner (i.e. low stability). Stability feedback is provided during gameplay by the buddy closing its eyes when the stability condition is met. In order to encourage collaborative behaviour, bonuses appear when the plank is stable for a certain time (i.e. four seconds), in the form of stars worth three points instead of one point.

#### Motor control measures

The following game-independent motor control measures were computed directly from the grip-force signals. To remove noise and spurious artifacts, force data was forward-backward filtered using a 10th order low-pass Butterworth filter with a 5Hz cut-off. All measures were used to compare intra-subject motor control across task conditions (i.e. single-versus-dual player modes).

1. **Effort:** Estimated as the root-mean-square of filtered force, which takes into account both expected force bias and variation.
2. **Smoothness:** Computed as the spectral arc length (SPARC) of the first derivative of the filtered force data [29], which is a sensitive and robust measure of smoothness, e.g. for evaluating intra-subject task-differences during motor control experiments.

#### Questionnaires

Following completion of all games, subjects were provided with questionnaires that assessed their engagement and preference of single-versus-dual player modes (Appendix (A) Questionnaires). The engagement questionnaire, based on the Intrinsic Motivation Inventory (IMI) consisted of questions divided into three subscales: enjoyment and interest, perceived competence, effort and importance [30, 31]. Subjects graded their opinion, referring to either single- or dual-player modes, on statements (e.g. ‘I tried very hard on this game’) using a scale from 1 to 7 (1 = ‘completely untrue’, 4 = ‘neutral’ and 7 = ‘very true’). Healthy subjects were provided with five statements per subscale (15 statements in total); for patients this was reduced to two statements per subscale (6 statements in total). The second part of the questionnaire evaluated user preferences of each player mode, as well as being questioned on which player mode subjects felt they ‘put most effort in’, ‘were the most skilled’ and ‘were the most pressured’. A box for free text comments was also provided. Patients were also asked if they wanted to continue playing and for those answering ‘no’, were asked for their reason.

#### Statistical analysis

Non-parametric, paired statistical tests were used throughout e.g. Mann-Whitney U (MWU) for testing performance differences between single and multiplayer scores, because of relatively small sample sizes, non-Gaussian distributions of the variables of interest, and an intra-subject design. For comparison of questionnaire results between player modes, a two-way Friedman test for the IMI questionnaire (> 1 question per category) and MWU for the user-experience questionnaire (1 question per category), were used. Correction for multiple comparisons was made using the Bonferroni method. Spearman correlation coefficients (*ρ*) were computed to measure associations between variables (e.g. IMI subscales, scores, etc.). Standard errors were calculated on the correlational values using a bootstrapping method on the data (e.g. age vs. scores) and 10,000 random resamples [32]. This allowed p-values to be estimated (e.g. between game-modes) to elucidate on significant differences across correlations by taking the difference between the variables of interest and counting the number of samples above or below zero (multiplied by two for a two-tailed test). To highlight relationships (e.g. between single-player and multiplayer scores), best fit lines were computed using ordinary least squares.

## Results

### Participants

Table 1 gives an overview of the numbers and characteristics of participants involved in both the studies.

**Table 1:** Demographics and information for the healthy and patient experimental groups

	Healthy	Stroke survivors
Group size [nr. of subjects]	32	16
Age [years]	26.3 ±4.5	70.3 ±19.7
Gender [M/F]	23/9	10/6
Dominant hand [R/L]	32/0	15/1
Affected side [R/L]	n/a	7/9
FMUE [/66]	n/a	51.3±13.6
S-FM [/12]	n/a	9.3±2.7

### Healthy-healthy experiment

#### Performance analysis

**Fig. 3:**
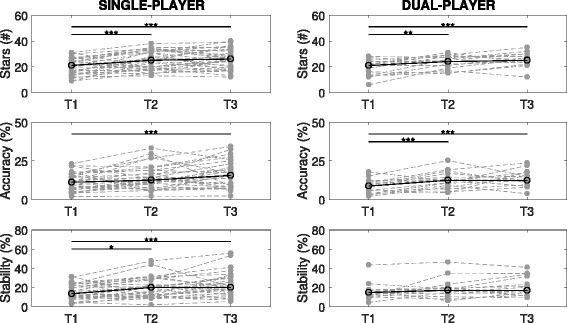
Healthy-Healthy Learning Effects. Scores during both single and dual-player modes over the three turns (T1, T2, T3) plotted for the three performance metrics: stars, accuracy and stability (* *p*<0.05; ** *p*<0.01; *** *p*<0.001)

A learning effect over three trials was seen in both single- and dual-player modes, across all three performance measures except during dual-player stability (Fig. 3; corrected for multiple comparisons). This learning effect occurred predominantly between trials T1 and T2 (or T3), whereas significant differences between T2 and T3 performance were never present. Therefore, the first trial was considered training, and trials T2 and T3 were pooled for further analysis.

There was no significant difference between single versus dual-player modes with regards to the number of stars collected (*p*=0.34) or stability (*p*=0.25). However, on average, accuracy decreased during the dual-player condition (15.1±7.9% vs. 13.0±5.3%; mean ± std; *p*<0.05). For each trial, the maximum number of stars that could be collected was 40 (including bonuses).

**Fig. 4:**
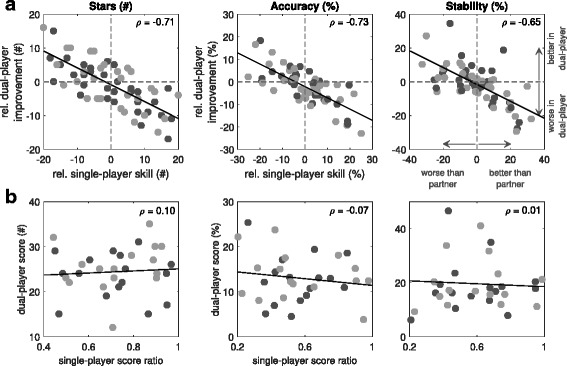
Healthy-Healthy Performance Measures Analysis. Comparison of single- and dual-player performances as a function of individual relative ability: **a** The relative improvement during multiplayer is positively correlated to the relative skill (i.e. difference in performances) of the other partner during their single-player turns. **b** The dual-player score does not depend on the individual performance ratios (worst divided by best player). The different circle colours highlight the different turns (T2 - light gray and T3 - dark gray)

To test whether there was a differential effect of game-mode across dyadic members, in terms of their individual skill levels, the relative effect of dual-player versus single-player mode for each member was compared with the difference in performance of the two partners during single-player mode. Figure 4 a highlights correlations between relative single-player skill and relative dual-player improvement across all three performance metrics (*p*<0.001; in all cases), such that the better a subject’s (single-player) performance, relative to their partner, the greater the drop in performance when jointly playing with them. Conversely, the worse a subject’s (single-player) performance, relative to their partner, the greater the improvement seen during dual-player gameplay.

Regardless of the mode of interaction, meaningful engagement in many inter-personal activities e.g. tennis, chess, depends upon ability matching. Therefore, performance as a function of partner disparity (in terms of their individual performances) was analysed. Figure 4 a highlights that there is no tailing off in the (linear) relationship between partner disparity and dual-player benefit at higher disparities (e.g. that would otherwise be seen as a sigmoid or other non-linear shape). This suggests that our paradigm offers the greatest gains for the poorest performers (when playing with the most skillful players). Figure 4 b reinforces this result by showing that there is no association between absolute dual-player performances and partner-mismatch (the latter measured as lower worse-to-better partner score ratios; stars: *p*=0.58; accuracy: *p*=0.68; stability: *p*=0.97). This suggests that the collaborative task is robust across a wide range of partner mismatches. Similarly the control of one player was perturbed in the dual-player game (trial T4), thereby increasing difficulty asymmetrically within a partnership. This was found to significantly deteriorate performance in terms of stability only, but not star collection or accuracy (Appendix (B) Healthy-healthy perturbation results).

**Fig. 5:**
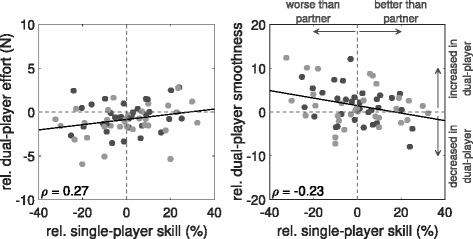
Healthy-Healthy Motor Control Analysis. Comparison of single- and dual-player effort and control smoothness as a function of relative individual ability: **a** Relative improvement of effort during dual-player is positively correlated to the relative skill (i.e. difference in stability performances) of the individual during the single-play mode (*p*<0.05). **b** Conversely, the relative improvement of control smoothness in dual-play is negatively correlated to the relative skill between the partners (*p*=0.069) although insignificant

Effort and control smoothness were compared across game modes as a function of partner disparity (Fig. 5; stability only). The results highlight that playing with a better partner significantly reduces the effort for the worse performing partners and vice versa for the better partner (*ρ*=−0.27,*p*<0.05). Conversely, smoothness generally improves in the more inferior partners, but worsens in the relatively superior players (*ρ*=−0.23,*p*=0.069), although the latter is an insignificant result. Nevertheless, this suggests that the compensation provided by the better partner allows poorer players to reduce their effort and focus a little more on their control.

#### Qualitative game assessment

**Fig. 6:**
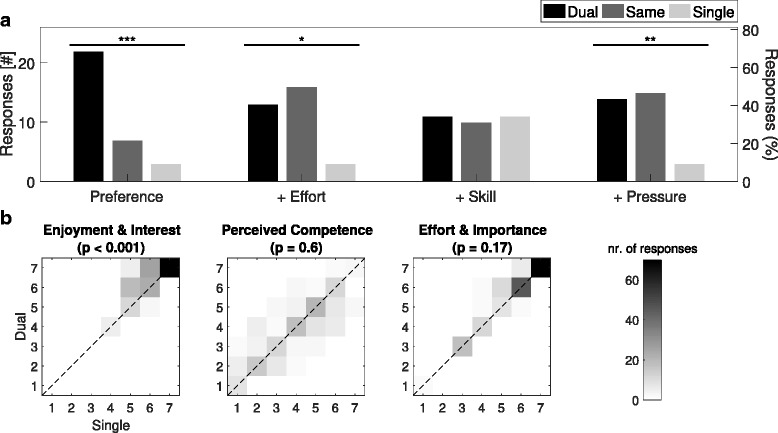
Healthy-Healthy Questionnaire Responses. **a** Histograms of user experience responses. **b** Joint distributions for single and dual-player responses to Intrinsic Motivation Inventory statement categories (^∗^ *p*<0.05,^∗∗^ *p*<0.01,^∗∗∗^ *p*<0.001)

Subjects expressed a preference for dual-player mode (Fig. 6 a), with 22/32 participants (69%) favouring this condition, as opposed to only three participants (9%) preferring the single-player mode (*p*<0.01), with the remaining subjects indifferent. The main reason given for favouring dual-player mode was that this made the game ‘more fun and unpredictable’. At the same time, dual-player mode was perceived to increase pressure (*p*<0.01) and effort (*p*<0.05). There was no significant preference for either mode in terms of self-reported skill (*p*=1.0). Comments about the game were enthusiastic, e.g. the design was ‘original and fun’, ‘an interesting and motivating scenario’, and ‘I liked the visuals’. There were no significant differences in self-reported difficulty comparing single vs. dual-player modes (*p*=0.16; single-player: 7±1; dual-player: 8±2.5; out of 10; median ± interquartile range). A positive correlation was present between the perceived player competence and actual single-player scores (stars: *ρ*=0.49, *p*<0.01; accuracy: *ρ*=0.56, *p*<0.001; stability *ρ*=0.51, *p*<0.01), but not between the perceived player competence and dual-player scores (stars: *ρ*=−0.07, *p*=0.71; accuracy: *ρ*=−0.04, *p*=0.82; stability: *ρ*=−0.04, *p*=0.82). No significant correlations were found between perceived effort or pressure, and the (single or dual-player) scores.

The IMI was answered more positively, in terms of Enjoyment & Interest, during dual-player mode (*p*<0.001; Fig. 6 b). However, there was no significant difference between game modes for Perceived Competence (*p*=0.60) or Effort & Importance (*p*=0.17). Correlations were also compared for the three IMI categories across the two gameplay modes. The only significant differences in correlation was found between Enjoyment & Interest and Perceived Competence (single-player: *ρ*=0.38, *p*<0.05; dual-player: *ρ*=0.007, *p*=0.97). Significant correlations were found between Enjoyment & Interest and Effort & Importance for both game modes (single-player: *ρ*=0.39, *p*<0.05; dual-player: *ρ*=0.51, *p*<0.01). No significant correlations were found between Perceived Competence and Effort & Importance in either game mode (single-player: *ρ*=0.27, *p*=0.13; dual-player: *ρ*=0.23, *p*=0.21).

### Patient-expert experiment

#### Participants

**Fig. 7:**
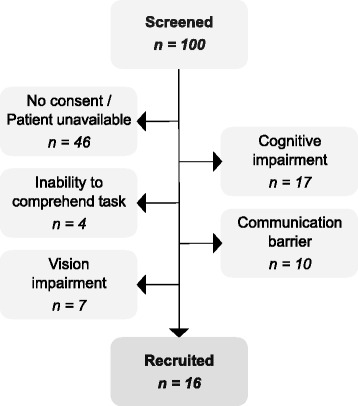
Patient Protocol. Reasons for exclusion in the cohort of stroke patients with arm weakness

One hundred consecutive stroke patients presenting with arm paresis secondary to acute stroke were screened. Figure 7 highlights the reason for patient exclusion leading to 16 subjects participating in the study. Table 1 provides characteristics of the recruited patients.

#### Performance analysis

Table 2 shows the average values (mean ± std) for the five metrics (stars, accuracy, stability, effort, smoothness) within the different game modes (single or dual) and trials, for both the healthy expert and patient players. No differences were found across the two trials (T1, T2), so the data was pooled across trials for subsequent analyses. As expected, all patients (using their paretic hand) performed worse than the healthy expert during single-player mode, with single-player scores (mean ±std) for patients (stars: 15.3±8.3, accuracy: 8.1±6.9%, stability: 8.1±8.3%) and expert scores across ten trials (stars: 44±0, accuracy: 87.6±10.6%, stability: 94.7±1.4%) significantly different (*p*<0.001 for all). The expert’s single-player score highlights that they were playing at a high and consistent level, especially the achievement in terms of the highest number of stars possible across all ten trials.

**Table 2:** Average values (mean ± std) for the five metrics (stars, accuracy, stability, effort, smoothness) within the different game modes (single or dual) and trials, during the patient-expert study (key: P - patient, E - expert)

	Expert single-player	Patients single-player		Patient-Expert dual-player	
	(10 reps)	T1	T2	T1	T2
Stars(#)	44±0	14.9±7.4	15.7±9.4	17.3±7.2	19.7±8.1
Accuracy (%)	87.6±10.6	7.7±6.1	8.4±7.9	9.6±5.8	11.2±8.5
Stability (%)	94.7±1.4	7.3±7.1	8.9±9.5	24.8±10.2	23.1±11.5
Effort (N)	10.8±0.9	10.2±4.4	8.4±4.1	P: 8.4±4.1	P: 8.2±3.6
				E: 13.1±1.6	E: 13.4±1.6
Smoothness	21.9±4.6	18.1±7.2	16.4±4.3	P: 15.4±3.4	P: 17.4±3.9
				E: 18.1±3.7	E: 19.0±4.5

**Fig. 8:**
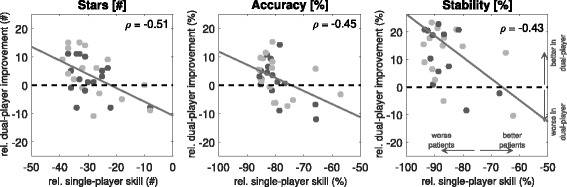
Patient-Expert Performance Measures Analysis. Effect of dual-player mode in patient-expert experiment for star-collection, accuracy and stability performance metrics. Relative improvement during dual-player interaction is seen for the majority of patients (especially in terms of stability). Furthermore, the greater the skill difference between patient and healthy expert, the greater the improvement afforded by the dual-player mode (stars: *p*<0.01; accuracy: *p*<0.05; stability: *p*<0.05)

Given that all patients were worse than the expert during single-player mode, and that dual-player mode benefited the inferior partner of healthy-healthy pairs, dual-player (patient-expert) mode was analysed in terms of patient performance relative to their single-player score. Figure 8 shows plots of dual-player improvement against (single-player) patient ability highlighting that not only did the majority of patients benefit from the dual-player mode (i.e. number and size of positive y data-points; stars: *p*<0.05; accuracy: *p*=0.07; stability: *p*<0.001; MWU test comparing game modes), but similarly to the healthy-healthy study, the extent of this improvement correlated with the extent of patient ability (Fig. 8; stars: *ρ*=−0.51,*p*<0.01; accuracy: *ρ*=−0.45,*p*<0.05; stability: *ρ*=−0.43,*p*<0.05). The performance measure in which the largest number of patients benefited from dual-player mode was stability. In some cases, stability rose from < 3% during single-player mode to nearly  40% during dual-player collaboration.

**Fig. 9:**
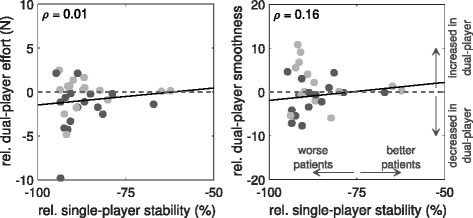
Patient-Expert Motor Control Analysis. Comparison of single- and dual-player effort and smoothness during patient-expert experiments: **a** Patient effort during dual-player, relative to single-player, mode is independent of patient stability during single-play (*p*=0.94). **b** Control smoothness during dual-player, relative to single-player, mode is independent of patient stability during single-play (*p*=0.37)

The effect of the game-mode on patient effort and control smoothness was examined. Figure 9 highlights the effect of relative ability compared to the expert’s performance on effort and smoothness (shown for stability only, as per analysis in healthy-healthy experiment). During single-player, patient effort was not significantly different to that of the healthy expert (9.3±4.3N vs. 10.8±0.9N, respectively; *p*=0.08; unpaired MWU test); whereas patients were inferior to the expert in terms of smoothness (17.2±5.9 vs. 21.9±4.6; *p*<0.01). Figure 9 shows that there is little association between patient performance and the effect of dual-player mode on effort or smoothness.

#### Relationship between impairment, age and performance

**Fig. 10:**
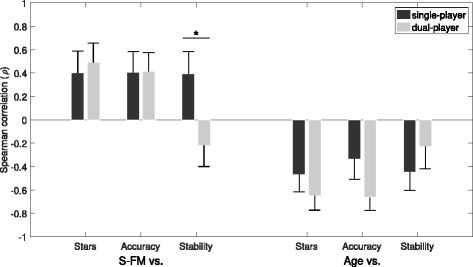
Patient-Expert Performance-Impairment Correlations. Comparison of single and dual -player game modes on Spearman correlations (*ρ*) between performance and general arm ability (Short-Fugl Meyer; S-FM), or performance and age, for each of three main performance metrics. Correlations are shown as mean and standard error calculated from 10,000 bootstrap samples. Based on these bootstrap distributions, differences between single and dual-player game modes in terms of correlation are also shown (* *p*<0.05 for S-FM vs. Stability only)

The effect of dual-player mode on patients, in terms of arm disability (rather than game performance) and age, was explored. Figure 10 shows that single-player performance across patients was, as expected, positively correlated with arm ability (Short-Fugl-Meyer; S-FM score), and negatively correlated with age, for all three performance measures. The effect of dual-player mode (i.e. interacting with an expert partner) was to slightly increase the magnitude of correlation for stars and accuracy, while decreasing it for the stability score. In the case of arm ability, the effect of dual-player mode was to significantly switch the correlation from positive-to-negative, suggesting that the expert provided proportionately greater (stability) compensation for patients with greater impairment (stars: *p*=0.71; accuracy: *p*=0.98; stability: *p*<0.05; tested using a nonparametric bootstrap method). For age vs. scores, there were no significant differences between single and dual -player modes across all the metrics (stars: *p*=0.34; accuracy: *p*=0.10; stability: *p*=0.36).

**Fig. 11:**
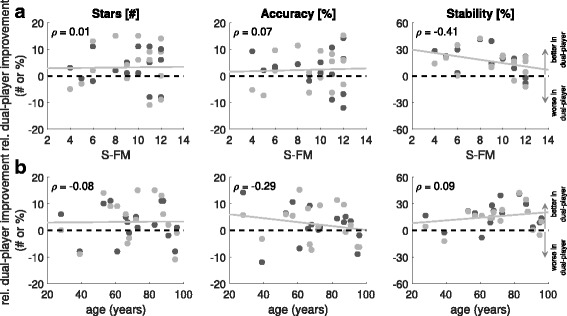
Patient-Expert Relative Performance. Relative dual-player improvement as a function of (**a**) general arm ability (S-FM) and (**b**) age, in patients, measured across the three scores

A related question is whether the relative benefits of dual-player mode amongst patients depended upon general arm ability and age. Figure 11 shows that dual-player improvement was found to correlate negatively with general arm ability, but only for stability (*ρ*=−0.41; *p*<0.05) and not for stars (*ρ*=0.01; *p*=0.94) or accuracy (*ρ*=0.07; *p*=0.69). For correlations of dual-player improvement with age, there were no significant correlations with dual-player improvement. However, a small negative trend (i.e. slightly greater improvement for younger patients) exists for accuracy (*ρ*=−0.29; *p*=0.11), while a possible positive trend for stability (*ρ*=0.096; *p*=0.64) appears to be weakened by subjects > 90 years old who benefit less from dual-player interaction. Dual-player improvement in the number of stars collected was independent of age (*ρ*=−0.08; *p*=0.65).

#### Qualitative game assessment

**Fig. 12:**
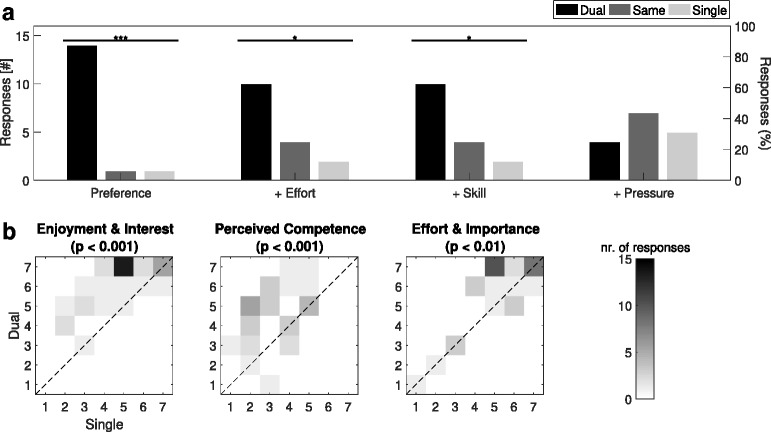
Patient-Expert Questionnaire Responses. **a** Histograms of user experience responses. **b** Joint distributions for single and dual-player responses to Intrinsic Motivation Inventory statement categories (^∗^ *p*<0.05,^∗∗^ *p*<0.01,^∗∗∗^ *p*<0.001)

Figure 12 a shows that similarly to the healthy subjects study, patients significantly preferred dual-player to single-player mode (88% vs. 6%; *p*<0.001) and felt that the dual-player mode allowed them to put more effort in (63% vs. 13%; *p*<0.05). Contrary to the healthy subjects, patients felt that dual-player interaction made them more skilled (63% vs. 13%; *p*<0.05), but not feel more pressured (25% vs. 31%; *p*=0.65). Typical participant comments included that dual-player mode was ‘more fun’, ‘motivating’, ‘easier with the guidance of an expert player’; ‘enjoyable, motivating and innovative’; and ‘the visuals were impressive’. Nine of the 16 (56%) participants wanted to continue playing that same day; while out of the remaining seven patients, a further five (31% of total patients) wished to play again on another day. Participants found the dual-player game significantly less difficult (4.5±3; median ± IQR; out-of-10) than the single-player mode (7±2.5; *p*<0.01). Figure 12 b also highlights that patients expressed significant preferences for dual-player mode in terms of all three categories (Enjoyment & Interest: *p*<0.01; Perceived Competence: *p*<0.01; Effort & Importance: *p*<0.001) of the IMI tested.

## Discussion

### Visual-coupling alone achieves an engaging zero-sum game

Results from the healthy-healthy study showed how collaboration influences the joint performance, by improving the score of the worse player while increasing the challenge for the better partner. Importantly, the difference in individual skill levels of the two players did not influence the performance, while interaction with a more skilled partner did not require more effort during dual-player interaction. The patient-expert study demonstrated that all patients were able to successfully play both the single and dual-player game modes regardless of impairment and age. Game performance was shown to dramatically improve during the dual-player mode, especially for the less skilled patients, with the expert partner providing support and compensation to keep the beam horizontal. Ultimately, this enabled the patients to collect more stars and be more accurate, while having a similar level of effort and smoothness. As expected, scores (i.e. stars and accuracy) generally got worse with impairment and age. However, the relative improvement (in terms of stars collected) during dual-player interaction showed no correlation with either impairment or age.

The results suggest that the joint performances are driven by an averaging process or zero-sum game based on the individual skill levels of the partners. By modifying the level of challenge experienced by each partner, they are more likely to achieve an appropriate ‘challenge point’, whereby the joint challenge and skill level are balanced (regardless of their individual skill levels) [33]. An appropriate challenge point is defined as the perception of engaging in challenges at a level appropriate to one’s capacity, and results in an intense focused concentration in the moment and a perceived sense of control over one’s actions [3, 4]. Moreover, the intrinsic skill balancing is not simply an averaging of the skill levels. Regardless of the ability of an individual, they would not succeed if playing with, for example, a (virtual) partner who was either completely constant or very noisy (i.e. very unskilled). This suggests that there is an operating band associated with the relative skill levels which permits a range of different skill levels to successfully play together while preventing either partner from completing the task alone.

Both healthy-healthy and patient-expert participants indicated a strong preference for the dual-player game over the single-player version. The dual-player mode increased enjoyment, perception of competence and self-reported effort amongst the patients, while giving them the sense of increased competence without increasing the pressure they felt. Therefore, individuals with and without sensorimotor limitations, found the collaborative game significantly more engaging than the single-player equivalent, and can be attributed to the skill balancing and social aspects that the multiplayer game affords.

### Collaborative gaming for physical training

The simplicity of the task implies that patients can also use the game to train with a variety of movements (e.g. grip, elbow flexion-extension, ankle plantar flexion-extension) and different rehabilitation devices. By increasing their engagement compared to playing alone, patients are more likely to increase the number of repetitions they perform and the effort they put into the training, which could ultimately lead to greater gains in performance [34, 35]. This game can be used in different rehabilitation scenarios involving, but not limited to, i) patients training with a therapist or relative, for instance a grandmother playing at home with her grandchild; or ii) patient-patient training e.g. whilst still at the hospital bedside or within community centers. Due to the low skill level of both partners in scenario (ii), the game parameters would (in some cases) need to be further adapted. In fact, by modifying different game parameters such as trajectory, background, speed, bonuses, obstacles etc., the game can be designed to incrementally increase the global challenge level for the dyad as their combined skill level increases with practise. For example, more difficult levels can be unlocked as the game progresses.

### Local redundancy balances the playing field and prevents slacking

The main focus of this multiplayer gaming concept is collaboration, promoting positive teamwork and social rehabilitation. Competitive games have been previously introduced [11, 13], where competition seemed to motivate some patients, but also discourage a significant proportion of them. However, the pong game used in these studies does not involve continuous interaction, in contrast to our game, and thus the results cannot be directly compared. Andrade et al. have previously developed a multiplayer rehabilitation game involving true interaction and collaboration [10]. The players receive haptic feedback providing additional information of the interaction, but have independent (orthogonal) control inputs so that an individual cannot help a patient to succeed in the task. This means that the game is not redundant, as one player’s action cannot compensate for a mistake from the other. In fact, it is the locally redundant nature of our interactive task that produces a challenging, but accessible exergame, for both partners independent of their relative skills, without requiring an additional skill-rating or skill-balancing algorithm (e.g. [36]). Previously, Vanacken et al. have introduced a ball-balancing concept utilising arbitrarily placed static targets, in contrast to a smooth continuous trajectory which is used in this study [23]. Our analysis has shown that, beyond just collaborative interaction, individuals of different skill levels can play together continuously (and without slacking), ultimately enjoying it more than single-player mode and suggesting that participants are more likely to exercise longer during dynamic multiplayer collaboration.

Local redundancy is achieved by ensuring that the players’ control inputs are non-orthogonal so that the actions of one player can compensate for the incorrect actions of another player, i.e. the control inputs are redundant. However, fully redundant inputs can easily lead to slacking behaviour, as either of the inputs can perform the actions for the other player and human motor control naturally minimises effort [22]. Therefore, the two inputs are ‘physically’ coupled (in the virtual world) so that only through active control from both inputs can it ensure that the end-effector (e.g. the buddy) can be moved correctly (without allowing the buddy to fall). Furthermore, defining the interaction in this manner allows the dyad to employ different strategies to complete the task. For example, during patient-expert collaboration, the expert seems to concentrate on minimising the buddy falling by increasing stability (i.e. by following the movements of the patient).

### Relation to actual physically-coupled paradigms

The collaborative rehabilitation games outlined in this paper are related to recent work investigating sensorimotor interaction in humans [16, 17, 37–39]. In [16, 17], pairs of subjects were connected by a virtual, but physically rendered, elastic band providing additional haptic information to the partners. The elasticity of the connection, meant that the partners could not rely on each other in order to succeed, implying neither could slack, a similar property elicited by this game. In contrast to our task, the partners could also work independently, which would not be adequate for neurorehabilitation where the patient requires assistance to move or control their limb. Both worse and better partners improved performance in [16], which is likely due to the additional haptic communication [17]. It would be interesting to study whether haptic feedback in combination with local redundancy could enhance the social rehabilitation experience further. However, using sensor-based technology (without active haptic feedback) provides decentralised therapy tools that are affordable and can be used both in-hospital and at-home. By combining these tools, such as the grip-force sensor used in this study, with socially engaging gaming concepts, patient engagement can be increased, ultimately leading to better patient compliance during rehabilitation.

### Limitations of the current study

The lack of a reactive single-player mode, e.g. based on an intelligent agent, could also be a factor as to why subjects had reduced performance and preference during the single-player version. A reactive agent, that could perform compensatory behaviour similar to the expert partner, could potentially increase the joint performance and also adapt to the partner as their ability progressed [16, 17]. Whether a patient would prefer playing with a human partner or computer agent would need to be further explored alongside any performance gains, which was beyond the scope of this study. Another limitation of the current study, necessary due to practical considerations, is the limited number of trials performed. This meant that patient motivation over longer training times could not be explored. Therefore, the next steps would be to explore the effect of our collaborative task during a longer motor learning paradigm involving healthy subjects and patients to see if (a) more efficient learning occurs and, (b) to examine if patients are more motivated to train for longer periods. We will also explore in more detail social aspects of interaction (e.g. conversation, playing with a relative vs. stranger, etc.) which are important to both performance and motivation [13, 40]. For instance, we will explore conditions where the dyads are either permitted or prevented to communicate during interaction and analyse the effect on their performance and qualitative evaluation. Beyond conversation, complete blinding of the participants to the gender, age, and demographic of their partner would also be an interesting avenue to explore.

## Conclusion

We have presented a framework to develop truly collaborative multiplayer gaming enabling two players to train together. The framework allows for the development of games that are simple, dynamic and interactive. A central property of the concept is its local redundancy, enabling players to help each other to succeed at the task, without replacing each other’s action entirely. This forces them to actively participate concurrently. Results from our healthy-healthy and patient-expert experiments highlight that: i) due to local redundancy, the game and scores can be modulated by the more skilled partner, although only within the bounds that the impairment or skill of the weaker partner allows, and ii) neither partner is able to ‘slack’ regardless of impairment, age and differences in relative skill levels. In the future, we expect new ‘collaborative gaming for physical training’ concepts to be developed, based on this simple framework, with the aim of increasing motor learning efficiency and patient engagement during virtual therapy tasks. The exploration of human-like agents during human-computer interaction scenarios alongside increased training times and the influence of social aspects, and their effect on performance, long term motivation and motor transfer, should also be studied.

## Endnotes

^1^ Note that the interactions in many previous studies are denoted as collaborative or cooperative. However the tasks specified in these studies do not require an exchange of information between the partners, e.g. in the Pong game, only an individual working alone is required to return the puck at any point in time [6, 11, 13]. Relative to Jarrassé et al.’s comprehensive taxonomy of interactive behaviours [14] these definitions have been used inconsistently and should actually be classified as co-active interaction.

^2^ Despite the many social implications of such a collaborative virtual task, examining social elements such as the effect of verbal communication was not considered during this study.

^3^ This force-to-height mapping is implemented using the Unity ^Ⓒ^ ‘SmoothDamp’ function.

^4^ A short video showing the game being played by a patient and therapist is provided as Additional file 1.


Additional file 1: Video showing the Balloon Buddies™ game being played by a patient and therapist. (MOV 73318 kb)


## Appendix

### (A) Questionnaires

#### Game experience

The following questions were asked to assess the experience of the players during the game and compare the single and multiplayer modes: QD Rate the difficulty of the 2 game conditions, from 0 to 10. QP Which game condition did you like the most? [Single, Same or Multi], Why? QE Which game condition did you put the most effort into? [Single, Same or Multi] QS During which game condition did you feel the most skilled? [Single, Same or Multi] QP During which game condition did you feel the most pressured? [Single, Same or Multi] QC Any comments (e.g. gameplay, feedback, visuals)?

#### Intrinsic motivation inventory

The following questions were asked to assess the intrinsic motivation during both game conditions (single and multiplayer):

1. I tried very hard on this game [P]
2. I think I am pretty good at this game [P]
3. This game was fun to play [P]
4. I did not put much energy into this
5. This game did not hold my attention at all
6. I was pretty skilled at this game
7. I thought this was a boring game [P]
8. I am satisfied with my performance at this game
9. I put a lot of effort into this
10. I enjoyed playing this game very much
11. I did not try very hard to do well at this game
12. This was a game that I could not play very well [P]
13. I thought this game was quite enjoyable
14. It was important to me to do well at this game [P]
15. I think I did pretty well at this game, compared with the others

*Note:* [P] indicates the questions selected in the patient version. This questionnaire uses a Likert scale from 1 to 7 (1= not at all true, 4= somewhat true and 7= very true)

### (B) Healthy-healthy perturbation results

**Fig. 13:**
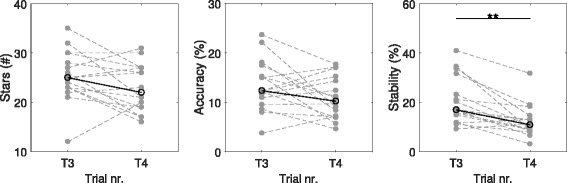
Healthy-Healthy Perturbation Results. Highlighting the difference in performance between turns T3 and T4 due to the perturbation introduced to the control of one of the partners prior to T4 during the healthy-healthy experiment. Only the stability metric shows a significant decrease (** *p*<0.01)

Figure 13 highlights the change in multiplayer scores between turn T3 (normal control) and T4 (perturbed control). Using a MWU test, it can be seen that only the stability metric decreases significantly. For the perturbed controller, a small increase of force now results in a larger change in the balloon’s height implying that balancing the beam is indeed harder to achieve. Moreover, the players were surprised by the sudden sensitivity and needed some time to adapt to this new control. Besides this effect on the stability, the performances were overall conserved, indicating that although the stars and accuracy scores decrease, the players still manage to collaborate efficiently. This strengthens the hypothesis that partners with different abilities are able to play together. For instance, a patient who experiences difficulties producing smooth movements (e.g. due to spasticity) may be helped by a healthy partner who can compensate for this.
